# Validating 3D Printing as a Rapid Prototyping Framework for Hemispherical Resonator: Design, Simulation, and Testing

**DOI:** 10.3390/s26123752

**Published:** 2026-06-12

**Authors:** Ali F. Abdulla, Jingning Ma, Mohamed Bognash, Samuel F. Asokanthan

**Affiliations:** 1 Department of Mechanical and Materials Engineering, The University of Western Ontario, London, ON N6A 5B9, Canada; aabdul54@uwo.ca (A.F.A.); jma588@uwo.ca (J.M.); or mohamed.bognash@uob.edu.ly (M.B.); 2 Department of Mechanical Engineering, Benghazi University, Benghazi P.O. Box 1308, Libya

**Keywords:** hemispherical resonator (HR), additive manufacturing, 3D printing, modal analysis, quality factor, PLA, vibration testing, Laser Doppler Vibrometer (LDV)

## Abstract

This paper investigates the viability of utilizing Fused Deposition Modeling (FDM) for the fabrication and follow-up testing of a hemispherical resonator (HR). This form of resonator has several significant applications, including the design of vibratory gyroscopes. While traditional high-precision resonators for this application rely on expensive fused-silica fabrication, this study proposes a macro-scale approach using Polylactic Acid (PLA) to enable accessible lab-scale experimentation. The specimens, featuring a unique central-hole mounting configuration, were designed in SolidWorks and analyzed via finite element methods to establish the modal hierarchy. Experimental Modal Analysis (EMA) was conducted using a Laser Doppler Vibrometer (LDV) to acquire vibration signals, which were then analyzed in NVGate, MATLAB, and MEscope to extract natural frequencies and quality factor. Results for a lab-scale HR specimen identified the n = 2 wine-glass mode with a deviation from theoretical natural frequency predictions largely attributed to inherent defects in the fabrication process. Furthermore, a frequency split of 2.15 Hz was observed due to the inherent asymmetries and mass imbalances of the fabrication method. The quality factor was evaluated via the ring-down method and validated using the half-power bandwidth (HPBW) technique. This work demonstrates that 3D-printed resonators serve as an effective, low-cost platform for isolating modal behaviors and optimizing geometric parameters before advancing to micro-scale fabrication.

## 1. Introduction

Gyroscopes are essential sensors for measuring angular velocity, serving as the backbone for navigation, guidance, and stabilization systems in the aerospace, automotive, and robotics sectors. Particularly, Coriolis Vibratory Gyroscopes (CVGs) have emerged as a prominent alternative due to their simplified mechanical structure, high sensitivity, and low power consumption. Within the CVG family, which employs structures such as beams, disks, rings, and shells, the Hemispherical Resonator Gyroscope (HRG) is widely considered the most accurate. These sensors utilize a thin, symmetric shell resonator that is maintained in a state of primary resonance. When the structure is subjected to angular rotation, the resulting Coriolis force causes a proportional transfer of energy into a secondary vibration mode, generating measurable signals that quantify the rate of rotation [1,2].

HRG has gained significant recognition in inertial navigation due to its exceptional reliability and long service life. For instance, high-end HRGs have demonstrated low bias stability, making them the sensor of choice for high-grade applications such as planetary exploration and oil borehole navigation [3]. This superior performance is largely attributed to the high symmetry of the hemispherical structure, which ensures minimal frequency mismatch and high insensitivity to environmental vibrations. Singh et al. [4] presented a fused-silica precision shell integrated micro-gyroscope that uses an out-of-plane transduction mechanism to achieve near navigation-grade performance. The 5 mm resonator demonstrated an impressive angle random walk of 0.0062°/h and a bias instability of 0.027°/h.

A critical performance indicator for these resonators is the Quality factor (Q-factor), defined as the ratio of stored energy to dissipated energy. A high Q-factor is essential for medium to high-precision applications as it improves the signal-to-noise ratio and reduces power consumption. However, achieving a high Q-factor is restricted by various energy dissipation mechanisms, including air damping, thermoelastic damping, and anchor loss [5,6,7]. In particular, surface and anchor losses are dominant sources of error, often stemming from manufacturing and material defects. Experimental measurements conducted by Zeng et al. [8] showed that post-fabrication treatments, specifically annealing and chemical etching, led to a measurable improvement in the resonator’s Q factor. Ma et al. [9] proposed a nonlinear regression model that links subsurface damage depth to surface roughness, highlighting that surface layer cracks significantly impair the performance of hemispherical resonators. The research concludes that achieving high-performance manufacturing requires the complete removal of subsurface damage, leading to a Q-factor improvement of several orders of magnitude. Pan et al. [10] reported the development of a monolithic cylindrical fused silica resonator that achieved a record-breaking Q factor approaching 8 × 10^5^. By leveraging the low internal friction and high isotropy of fused silica, their 25 mm diameter resonator demonstrated a ring-down time exceeding one minute, marking a significant advancement for cylindrical vibratory gyroscopes.

While fused silica is often preferred for its low internal friction and exceptionally high Q-factors, its complex manufacturing process and high production costs limit its accessibility for rapid prototyping and exploratory experimental research [11]. To address these manufacturing challenges, 3D printing offers a promising technology for resonator fabrication, provided that the inherent limitations of the process, such as material damping and structural anisotropy, are carefully accounted for during the design phase. Additive manufacturing facilitates the direct translation of digital CAD models into physical parts via layer-by-layer deposition, enabling the decentralized production of complex, customized geometries [12,13]. Techniques such as Fused Deposition Modeling and Stereolithography allow for the production of complex, customized geometries with integrated components, significantly improving manufacturing efficiency and reducing costs [14,15]. Hou et al. [16] introduced the first 3D-printed micro hemispherical shell resonator, fabricated via high-precision projection micro stereolithography to bypass complex traditional MEMS manufacturing steps. The resulting device features tunable resonant frequencies and high-quality factors in air, demonstrating a path toward rapid batch manufacturing of high-performance resonators for portable navigation. Despite challenges with limited material options and reduced mechanical strength compared to traditional methods, recent research on composite materials and post-processing suggests strong potential for 3D-printed devices. It suggests that the adoption of 3D printing is rapidly expanding due to its ability to simplify assembly and reduce overall structural complexity and fabrication costs [17].

While macroscale HRGs have proven their excellence, there is a growing demand to minimize their cost, size, weight, and power (CSWaP) through miniaturization. However, maintaining structural symmetry and material properties at smaller scales remains a significant hurdle, as material defects and residual stresses can severely impair resonator quality [11]. This research investigates a novel lab-scale approach to resonator development by utilizing macro-scale 3D-printed Polylactic Acid (PLA) hemispherical structures for dynamic characterization. The methodology focuses on the precise measurement of the quality factor and mode shape analysis using a specialized experimental setup. This approach serves to validate 3D printing as a viable, low-cost tool for the rapid prototyping, design, and iterative testing of next-generation resonators. Although advanced volumetric printing methods seek to eliminate layering artifacts, they struggle with structural scalability for macro-scale components [18]. This study explicitly embraces the challenges of layer-by-layer extrusion, aiming to test the practical operational limits of standard FDM pipelines and evaluate how manufacturing-inherent features, such as the toolpath Z-seam, influence the underlying structural dynamics of hemispherical shell resonators.

A significant contribution of this work lies in the use of macro-scale prototypes to bridge the gap between theoretical modeling and high-precision manufacturing. By scaling up the resonator geometry, this study allows for a more accessible and detailed observation of modal behaviors and energy dissipation mechanisms in this class of resonators that are often difficult to isolate at the smaller sizes.

## 2. Structural Design and Methodology

The calculation of the natural frequency for the hemispherical resonator in this study is based on the theoretical framework established by Huo et al. [19]. The modeling process begins by deriving geometric deformation equations based on the Kirchhoff-Love hypothesis within the theory of elastic thin shells. By applying the vibration mode and the stress–strain relationship of a hemispherical thin shell, the deformation energy equation is formulated. Furthermore, the kinetic energy is determined by incorporating the Coriolis effect to account for the rotation. These energy components are integrated into Lagrange’s principle of mechanics to derive the comprehensive motion equations of the resonator. From these equations, the natural frequency and precession factor are calculated by solving the characteristic equations of the vibration system.

Drawing on the theoretical framework established by Alahakoon, this study prioritizes optimizing frequency separation to ensure modal stability [20]. As noted by Mayorca et al. [21], minimal frequency separation can lead to mode instability, which compromises gyroscopic precision. To mitigate the risk of energy transfer between the dominant flexural frequency (DFF) and neighboring modes, a design objective of 25% frequency separation was targeted. The current model incorporates specific structural reinforcements intended to maximize this separation. This theoretical foundation guided the selection of the shell thickness and mounting hole configuration, ensuring that the n = 2 wine-glass mode remains sufficiently isolated from adjacent parasitic modes during experimental excitation.

To ensure the accuracy of the proposed model, the calculated natural frequencies were validated by comparison with numerical simulations using the finite element approach and experimental measurements. The theoretical values derived from the governing equations of motion showed high consistency with the finite element analysis, demonstrating that the analytical model can effectively substitute for FEA simulations when computational resources or software are unavailable. However, both numerical approaches exhibited a quantitative deviation from the initial raw experimental data, establishing a baseline error bound that is explicitly addressed and reconciled through physical manufacturing parameters in Section 5. Additionally, the model successfully captured the varying trends of natural frequency in response to changes in the physical and geometrical parameters of the resonator, mirroring the behavior observed in the finite element analysis. These results confirm the correctness and rationality of the motion equations as a reliable foundation for analyzing the dynamics of the hemispherical resonator.

The structural performance of a hemispherical resonator is fundamentally dictated by its geometric precision and the strategic placement of mounting features. The hemispherical resonator geometry was developed in SolidWorks, moving away from traditional stemmed designs in favor of a central mounting hole configuration. This design choice allows for a more direct coupling to the test base while facilitating the study of how localized geometric features influence modal behavior. The CAD model was constructed using revolving features to define the primary shell, followed by precise cut-extrude operations to incorporate the central mounting point and a series of eight smaller peripheral holes distributed around the center. These parameters, specifically the shell thickness, the primary hole diameter, and the configuration of the surrounding small holes, were identified as the critical design variables that dictate the natural frequency separation and the resulting modal order.

This specific geometric arrangement was required to achieve a reasonable initial separation between the degenerate modes, ensuring that the n = 2 “wine-glass” mode could be isolated and analyzed without interference from adjacent structural resonances. Following the design phase, the model was subjected to a finite-element modal analysis in SolidWorks. By applying the material properties of Polylactic Acid (PLA) summarized in Table 1, the simulation predicted the resonator’s dynamic characteristics, confirming that the chosen hole patterns achieved the desired modal frequency order as well as separation before proceeding to physical fabrication.

**Figure 1:**
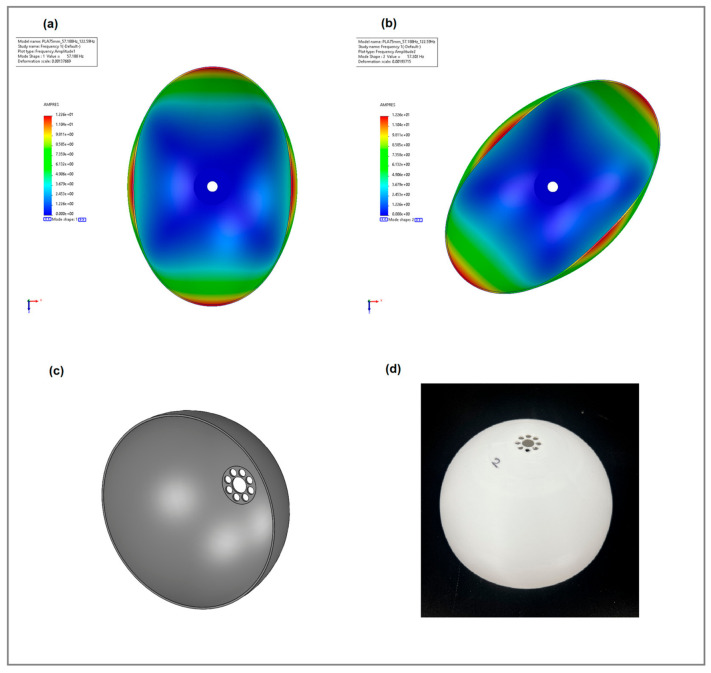
The second flexural mode is presented via SolidWorks simulation: (**a**) Primary mode; (**b**) Secondary mode; (**c**) CAD model of the hemispherical shell resonator; (**d**) Top-down view of the fabricated prototype sample 2.

The modal hierarchy of the hemispherical resonator differs significantly from that of cylindrical structures, often resulting in the second flexural mode n = 2 appearing as the primary resonance. For the current design, characterized by a specific shell thickness and a central mounting hole, the mode shapes and their respective orders were computed to establish a baseline for experimental comparison. In this configuration, the wine-glass mode is the primary detectable response, manifesting as two degenerate modes, known as the primary and secondary modes, with natural frequencies of 57.18 Hz and 57.2 Hz, respectively. These modes are physically oriented at 45°, as illustrated in the simulation results in Figure 1a,b. Additionally, the corresponding CAD geometry and physical prototype are shown in Figure 1c,d.

For fabrication, the CAD model developed in SolidWorks was exported to the Dremel Digilab 3D Slicer. The specimens were manufactured using a Bambu Lab (P1P/X1C) system (Bambu Lab, Austin, TX, USA) equipped with a 0.4 mm nozzle, utilizing a layer height of 0.2 mm to balance print resolution and structural integrity. The hemispherical shell resonator was fabricated utilizing Bambu PLA filament at a nominal extrusion temperature of 220 °C. The specimen was printed in an apex-up orientation utilizing two perimeter wall shells, five top layers, and three bottom layers. The internal cavity was specified with a 20% infill density utilizing a straight-line filling strategy. Temporary tree supports were implemented during the build cycle and subsequently removed by post-fabrication. The part was printed directly onto the build plate without the use of auxiliary adhesion layers.

Post-fabrication metrology was conducted using standard digital calipers and micrometers for thickness and a printed centimeter grid paper for diameter to evaluate the manufacturing tolerances and dimensional fidelity of the printed specimens against the nominal CAD design. The physical parameters, including the outer diameter and wall thickness yielded average post-printed structural dimensions of 149 mm and 1.03 mm, respectively, indicating minor thermal shrinkage inherent to the cooling profile of the FDM process.

In summary, an iterative analytical design process was used before fabrication to ensure the structural parameters matched the target performance specifications. This framework offers a high-precision predictive tool that effectively reduces the computational load often seen in finite element modeling. By allowing quick estimation of resonant frequencies directly from geometric variables, this model simplifies the initial design stage and supports efficient prototyping of 3D-printed resonators.

## 3. Experimental Setup

**Figure 2:**
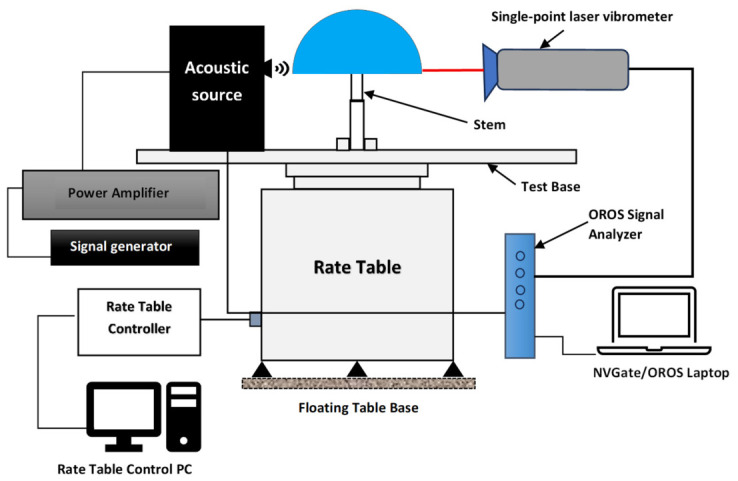
Schematic representation of the experimental setup showing the integrated sensing and actuation system.

The experimental arrangement was designed to isolate the resonator from environmental noise and accurately capture high-frequency vibrations. A comprehensive overview of the experimental arrangement and detailed schematic representations is provided in Figure 2. In order to ensure high-fidelity characterization of the 3D-printed resonators, the experimental setup was designed with precision and to provide necessary noise mitigation. The entire apparatus is sitting on a floating table base that serves as a vibration-isolation system, effectively eliminating floor-borne dynamic interference and external environmental noise. For Experimental Modal Analysis (EMA), the resonator was rigidly secured via its central mounting hole using a steel bolt and dual washers to establish a reproducible clamped boundary condition. The specimen was bolted directly to an aluminum fixture base, whose mass is significantly higher than that of the PLA shell, to ensure excellent mechanical impedance matching and minimize parasitic energy leakage into the support structure, without the use of adhesives or elastomeric dampers. For the purpose of actuating the non-metallic PLA shell, acoustic excitation was selected to provide a purely non-contact energy transfer, thereby avoiding the mass-loading effects associated with attached actuators [22]. A speaker, driven by a Meterman FG3C function generator (Wavetek, San Diego, CA, USA), is strategically positioned at an anti-nodal line of the structure to excite the free hemispherical end.

Sensing is achieved via a Laser Doppler Vibrometer (LDV), model No: OFV 303.8 by Polytec GmbH in Waldbronn, Germany, which provides high-accuracy, non-contact measurements of the shell wall velocity and displacement. This optical sensing method is particularly ideal for polymer structures like PLA, where traditional metallic coatings required for electrostatic sensing might alter the resonator’s mass distribution and symmetry. For optical signal acquisition, a small patch of reflective tape was applied to the resonator’s circumferential edge to enhance the laser beam backscatter. A Crown CE1000 amplifier by Crown Audio Inc., Elkhart, IN, USA, was used to drive the excitation signal, with responses recorded via a National Instruments DAQ system. Data acquisition and initial spectral identification were performed using NVGate version 14 and an OROS (OR35) signal analyzer by OROS Americas Inc., in Grand Haven, MI, USA. This was followed by post-processing in MEscope version 22.0.1.27 to conduct a comprehensive modal analysis. This dual-system approach ensures the precise extraction of natural frequencies, damping ratios, and mode shapes required to quantify the quality factor.

The integration of the hardware components was managed via NVGate interface that synchronized the excitation sweep with the data-logging process. During characterization, the function generator produced a sinusoidal sweep signal across a broad frequency range to locate the primary resonant peaks, after which a narrow-band zoom analysis was performed to isolate the n = 2 wine-glass mode with high spectral resolution. The OROS analyzer processed the incoming LDV signals through a Fast Fourier Transform (FFT) algorithm, enabling real-time monitoring of the Frequency Response Function (FRF). To validate the consistency of the damping measurements, the time-domain decay data captured during ring-down testing is cross-referenced with the frequency-domain half-power bandwidth results. This rigorous data processing ensured that the extracted modal parameters were independent of experimental artifacts, providing a reliable basis for evaluating the quality factor of the 3D-printed specimens.

## 4. Mathematical Framework for Q-Factor Estimation

The Quality factor (Q) is the primary metric for evaluating energy dissipation in the resonator. The literature typically categorizes Q-factor measurement into three primary frameworks: frequency, time-frequency, and time domains [23]. To evaluate energy dissipation in this study, the time-domain ring-down (free decay) method and the frequency-domain half-power bandwidth approach have been employed for cross-validation and accuracy.

### 4.1. Time-Domain Decay Analysis

The free vibration of a damped harmonic oscillator, once the excitation source (acoustic impulse) is removed, is governed by the following displacement equation in the time domain:

(1) $$X\left(t\right)=Ae^{\left(-\zeta \omega_{n}t\right)}Cos\left(\omega_{d}t+\phi \right),$$

where $\omega_{n}$ and ζ , respectively, represent the natural frequency and the damping ratio while $\omega_{d}$ denotes the damped natural frequency. The arbitrary constants A and ϕ depend on the imposed initial conditions. The temporal evolution of the oscillation amplitude is governed by the exponential envelope $e^{-\zeta \omega_{n}t}$. The decay time τ is defined as the characteristic interval required for the amplitude to decay to 1/e of its initial value A. Comparing the response at time t and t+τ, the following fundamental reciprocal relationship can be established:

(2) $$\tau =1/\left(\zeta \omega_{n}\right)\;.$$

The Quality Factor (Q) characterizes the damping of the resonator relative to its natural frequency, traditionally defined as $Q=1/\left(2\zeta \right)$. Hence, using Equation (2) the quality factor Q is expressed in terms of the experimentally observable decay time as

(3) $$Q=\left(\tau \omega_{n}\right)/2\;.$$

Further, Equation (3), when the natural frequency $\omega_{n}$ is expressed in Hz, becomes

(4) $$Q=\pi \omega_{n}\tau \;.$$

The mathematical framework established above served as the governing logic for the MATLAB-based analysis of the Single-Degree-of-Freedom (SDOF) system. Specifically, the exponential decay envelope, $e^{\left(-\zeta \omega_{n}t\right)}$, was utilized as the regression model for an iterative least-squares curve-fitting algorithm. By extracting the peaks of the experimental displacement data through a peak-detection routine, the code performs a nonlinear fit to isolate the time constant τ. Once τ is statistically converged, the algorithm computes the quality factor using the derived relationship in Equation (4). This approach ensures that the quality factor calculation is grounded in the global energy dissipation characteristics of the measured signal rather than a single-point measurement, thereby reducing the impact of experimental noise on the final damping estimation. This mathematical model forms the foundation for predicting quality factor results via the ring-down method.

### 4.2. Modal Extraction in the Frequency-Domain

The accuracy of modal parameter extraction depends heavily on the robustness of the analytical methods used to interpret experimental data. Cross-validation between different processing techniques is essential for ensuring that calculated damping ratios and resonant frequencies are true structural characteristics rather than artifacts of noise or signal processing errors. By employing a dedicated post-processing environment, experimental results can be rigorously verified against mathematical models to ensure high-fidelity characterization of the system’s dynamic performance. Experimental Modal Analysis (EMA) was conducted using MEscope to accurately characterize the dynamic behavior of the 3D-printed resonators. This software is a powerful post-processing suite designed to observe and analyze the vibration of mechanical structures by extracting modal parameters, such as natural frequencies, damping ratios, and mode shapes, from measured response data. It is widely utilized in Operational Modal Analysis (OMA) and EMA because of its ability to fit mathematical models to transient or steady-state waveforms. By applying advanced curve-fitting algorithms, the software enables the isolation of specific vibration modes from complex experimental data, which is essential for understanding how energy is dissipated within the system and for validating the structural integrity of the resonator.

The modal extraction process in this study followed a multi-step workflow focused on the n = 2 flexural mode of the hemispherical shell. Initially, the velocity signals captured by the Laser Doppler Vibrometer (LDV) were converted from the time domain into the frequency domain. This spectral analysis allows for the precise location of the n = 2 flexural mode and identifies any frequency splitting. Once the mode was identified, a polynomial method was applied to fit a mathematical model to the primary frequency peak [24]. This curve-fitting process employs a least-squares approach to solve for the best-fit values of the mode amplitude, damping ratio, natural frequency, and phase angle.

Measuring these modal parameters provides a comprehensive characterization of the 3D-printed resonator’s structural and functional performance. The natural frequency serves as a vital indicator of the stiffness-to-mass ratio, enabling direct comparison between experimental results and FEA simulations to assess the effective stiffness of the printed PLA. The damping ratio is equally significant, as it characterizes internal energy dissipation and provides the mathematical basis for calculating the quality factor (Q), which ultimately determines the sensor’s sensitivity. Meanwhile, monitoring amplitude and phase angle ensures the validity of the results; a 90° phase shift confirms that the system has reached a true resonant state rather than a localized noise peak, while amplitude tracking reveals the resonator’s responsiveness to acoustic excitation and its linear behavior under varying loads. However, the experimental modal analysis and characterization focused primarily on capturing time-domain decay via ring-down tests and tracking frequency-response amplitudes. Consequently, phase data were omitted from the experimental characterization. Together, these variables allow for a detailed mapping of how fabrication asymmetries and polymer material properties influence the dynamic stability of the n = 2 wine-glass mode. In this context, stability refers to the preservation of a consistent and symmetrical vibration pattern over time, ensuring that the modal characteristics do not drift or degrade due to fabrication-induced imbalances or material viscoelasticity. By minimizing the error between the measured response and the fitted decay, MEscope isolates the damping characteristics required for precise calculation of the quality factor.

## 5. Experimental Characterization Results and Discussion

To evaluate the dynamic accuracy of the 3D-printing process, the fabricated specimens underwent systematic frequency response characterization to correlate their physical performance with the initial computational models. Initial characterization of the resonator was conducted using a single-point Laser Doppler Vibrometer (LDV). Experimental frequency response data identified a fundamental natural frequency of 47.01 Hz, representing a significant downward shift from the simulated theoretical value of 57.2 Hz. This discrepancy is primarily driven by the physical realities of the Fused Deposition Modeling (FDM) process, where microscopic voids between extruded beads and weaker interlaminar bonding result in a lower effective structural stiffness than the idealized, isotropic CAD model assumes. Furthermore, the “staircase effect” from the 0.2 mm layer height and minor compliance at the 3D-printed mounting interface introduce additional mass and stiffness deviations, collectively lowering the observed resonant frequency. To evaluate manufacturing reproducibility, replicate testing was performed on a batch of three identically printed resonators (N = 3), yielding a mean experimental fundamental frequency of 48.87 Hz with a standard deviation of 1.55 Hz. The 95% confidence interval was found to be [45.021 Hz, 52.72 Hz], confirming high modal reproducibility and reducing the quantitative deviation from the analytical model (57.2 Hz) to a reasonable 14.6%.

**Figure 3:**
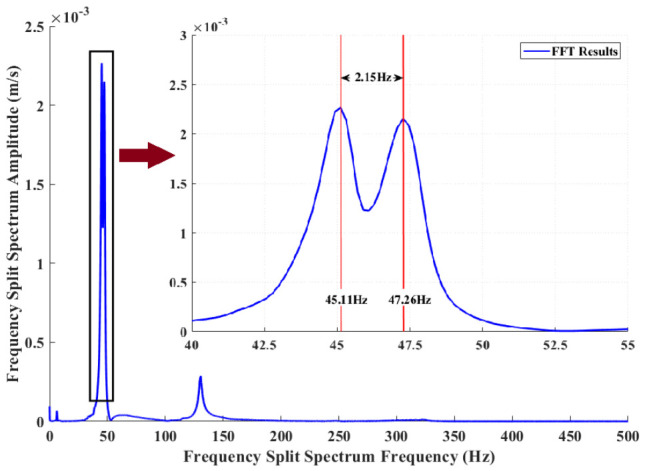
Frequency response of n = 2 wine-glass modes.

The frequency response of the 3D-printed hemispherical resonator was characterized at room temperature under atmospheric pressure. Using a signal generator for acoustic excitation, a frequency sweep was conducted and monitored via the NVGate program to identify the primary resonant peaks. To ensure data reliability, the acoustic actuator was locked at a fixed distance from the shell, and the excitation signal was driven by a constant-voltage amplifier. Preliminary tests confirmed that doubling and tripling the excitation amplitude yielded no measurable shift in natural frequency or damping, validating that the system operated strictly within its linear dynamic range. As illustrated in Figure 3, the n = 2 “wine-glass” modes were identified at 45.11 Hz and 47.26 Hz. The frequency split is primarily attributed to the FDM toolpath’s Z-seam, which introduces a localized mass imbalance and stiffness discontinuity along a single longitudinal axis of the hemisphere. This systematic asymmetry breaks the circumferential degeneracy of the structure, resulting in a frequency split of 2.15 Hz. This loss of symmetry causes the separation of the naturally degenerate modes, as further validated and evidenced by the peak separation directly observed in the MEscope results.

Following the identification of the resonant frequencies, the damping characteristics were quantified to evaluate the energy dissipation performance of the 3D-printed structure. For Q-factor measurement, the quality factor Q was determined through a standardized ring-down test conducted at room temperature [10,25]. The process began by exciting the resonator at its n = 2 resonant frequency until a steady-state response was achieved. Upon reaching peak amplitude, the excitation signal was abruptly terminated, allowing the structure to undergo free-vibration decay, which was captured in real time by the OROS analyzer system. The time constant (τ) and the corresponding quality factor was derived by applying an exponential curve fit to this envelope.

**Figure 4:**
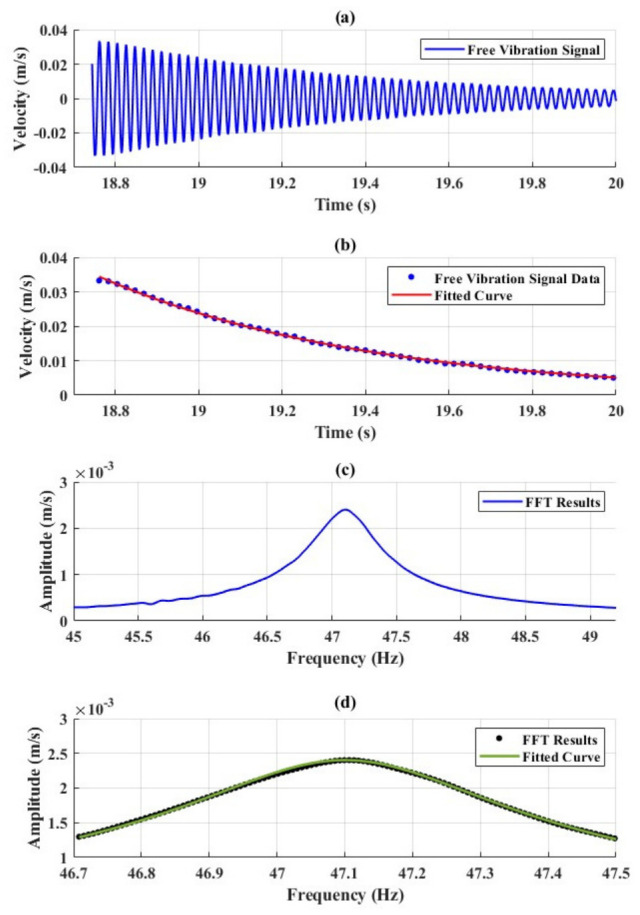
(**a**) Extracted time signal, (**b**) Time domain exponential decay. (**c**) Output FFT results. (**d**) Natural frequency peak in FFT results.

Figure 4a,b, represent the experimentally measured free-decay response as well as the results of the curve fitting process. The recorded signal was processed in MATLAB R2021a to extract the decay time and compute the corresponding damping ratio. Figure 4c,d, display the natural-frequency peak in the frequency domain for the second flexural mode of the resonator. The frequency-response data in this region were subsequently curve-fitted using a single-degree-of-freedom (SDOF) model to obtain a refined estimate of the damping ratio.

**Figure 5:**
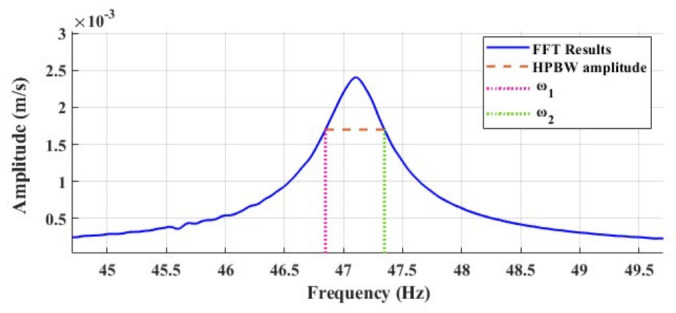
Half-Power Bandwidth Method results.

To provide a robust validation of the modal parameters, the experimental data were also analyzed using the half-power bandwidth method. This classical frequency-domain approach serves as an independent cross-check for the damping ratios and natural frequencies extracted via the initial curve-fitting techniques. As illustrated in Figure 5, the method involves identifying the resonant peak and calculating the frequency intervals $\omega_{1}$ and $\omega_{2}$ at which the response magnitude drops by a factor of 1/$\sqrt{2}$ of the maximum amplitude, which is equivalent to a 3 dB drop. By comparing the results from both the SDOF fitting modal and the half-power bandwidth calculations, the consistency of the damping measurements was confirmed, ensuring that the reported quality factors are not artifacts of a single processing algorithm.

**Figure 6:**
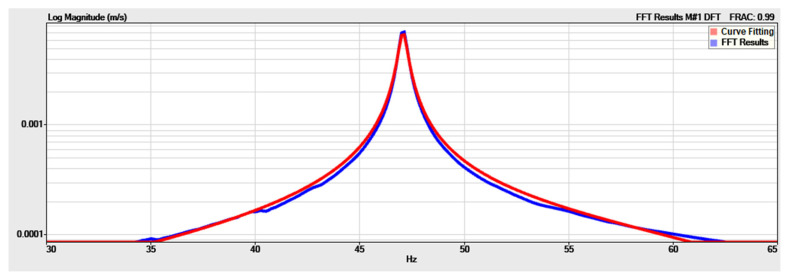
MEscope curve fitting and mode shape results.

To supplement the MATLAB results, the data were further analyzed using MEscope to verify the resonant characteristics and extract the damping properties of the structure. The raw time-domain signals captured by the LDV were processed within MEscope, where a Fast Fourier Transform (FFT) was performed to visualize the energy distribution across the frequency spectrum. As illustrated in Figure 6, this transformation confirmed that the structure resonates at a primary frequency of 47.07 Hz, providing a secondary validation of the resonant peak identified during the initial frequency response testing. This spectral consistency ensures that the subsequent damping analysis is performed exactly at the resonator’s natural frequency.

Following the FFT transformation, a curve-fitting technique was applied to extract the modal parameters. As shown in Figure 6, the curve fitting was targeted specifically within a narrow frequency band surrounding the 47.07 Hz natural frequency peak. By isolating the second flexural mode, the software precisely determined the damping ratio. The resulting damping ratio of 0.4129 for this structure provides a key data point for evaluating the energy retention capabilities of 3D-printed PLA in gyroscopic applications.

The modal parameters of the 3D-printed hemispherical resonator were extracted using time-domain ring-down (free decay) and the frequency-domain half-power bandwidth methods to ensure the reliability of the damping estimation. As summarized in Table 2, the natural frequency $\omega_{n}$ remained remarkably consistent across all methods, centering around 47.10 Hz. However, slight variations were observed in the quality factor (Q), depending on the processing software and the employed mathematical approach. While MEscope polynomial curve fitting yielded the highest Q of 121.1, MATLAB curve-fitting methods provided more conservative values using three distinct time-domain and frequency-domain approaches. The Exponential Decay Fit (Ring-down) yielded the highest Q factor of 98.65, suggesting it may be more effective at capturing the resonator’s intrinsic losses by avoiding the spectral leakage inherent in FFT-based methods [26]. In contrast, the Half-Power Bandwidth (HPBW) and FFT + SDOF Fit methods produced slightly lower values of 94.35 and 94.79, respectively. The close agreement between HPBW and FFT + SDOF fit suggests that, although 3D-printed imperfections introduce frequency splitting, the individual peaks still closely follow linear single-degree-of-freedom behavior for damping extraction.

The experimental results highlight the inherent trade-offs of employing FDM for the fabrication of high-frequency resonators. While the characteristic n = 2 “wine-glass” mode shapes were successfully achieved and verified using MEscope, the measured Quality (Q) factors are significantly lower than those of traditional fused-silica or metallic shells. Unlike the nearly perfect elastic behavior of fused silica, which minimizes thermoelastic damping, Polylactic Acid (PLA) exhibits significant viscoelasticity. This leads to higher internal friction, in which vibrational energy is rapidly converted into heat through molecular chain friction during each oscillation cycle [27,28]. In resonant structures, surface loss becomes increasingly dominant as the surface-to-volume ratio increases or surface quality decreases. In this case, the 0.2 mm fabrication layer creates a distinct “staircase” profile along the hemispherical curvature. This geometric roughness acts as a site for increased energy scattering and localized stress concentrations, which significantly reduce the Q factor compared to ground or polished surfaces [29]. The established testing framework can easily adapt to advanced post-processing optimization. Future work can explore the application of ultra-thin sputtered bimetallic coatings, a pathway recently demonstrated to significantly mitigate surface and thermoelastic dissipation in cylindrical resonators, as a means to enhance the precision of 3D-printed architectures [30].

## 6. Conclusions

This study validates the use of 3D-printed PLA structures for experimental characterization of hemispherical shell-based resonators. The integration of an analytical model into the design workflow ensures the precise delivery of desired modal characteristics while reducing reliance on intensive simulation. The ability to derive resonant frequencies directly from geometric parameters provides a robust, closed-form alternative to numerical modeling, significantly accelerating the development cycle for macro-scale hemispherical resonators.

By utilizing non-contact acoustic excitation and Laser Doppler Vibrometry, the natural frequencies and quality factors of the hemispherical resonator were successfully characterized. While PLA does not yet match the performance of high-grade navigation materials, the ability to rapidly iterate on designs using additive manufacturing opens new pathways to study complex resonator geometries before moving to expensive, high-precision fabrication.

The experimental characterization of the lab-scale 3D-printed resonator reveals a fundamental trade-off between manufacturing efficiency and resonator performance. While 3D printing technology successfully replicated the characteristic n = 2 mode shapes, the measured quality Q factors remained significantly lower than those achieved by traditional metallic or fused silica resonators. This performance gap is primarily attributed to the inherent viscoelasticity of the PLA material, which facilitates higher internal energy dissipation via molecular chain friction.

The structural integrity and performance of the resonators were primarily dictated by the additive manufacturing process, including the “staircase” effect and the print seam and nozzle transition points, which may have introduced mass imbalances that compromised the resonator’s symmetry. These geometric asymmetries resulted in observable frequency splitting in the MEscope data, preventing the perfect alignment of degenerate modes and impacting the stability of the gyroscopic signal. Despite these performance trade-offs, the study validates 3D-printed PLA as a viable, cost-effective platform for rapid lab-scale prototyping and the investigation of complex gyroscopic geometries. Future investigations will focus on conducting a comprehensive parametric study to systematically correlate the observed 2.15 Hz frequency split with deterministic manufacturing features, utilizing high-resolution 3D scanning to isolate localized geometric imperfections and mapping the precise orientation of the toolpath Z-seam relative to the modal axes. It should also focus on systematically manipulating print parameters, including layer height, print speed, and thermal extrusion profiles, as a logical mechanism for tailoring the quality factor and achieving precise modal tuning in polymer resonators.

The measured quality factor (Q) of the 3D-printed PLA is significantly lower than that of traditional fused silica benchmarks and may not yet meet the stringent material requirements for navigation-grade applications due to its high intrinsic dissipation. However, this study aims to test the current boundaries of the art by exploring the feasibility of additive manufacturing for complex resonator geometries. As 3D printing technology matures with the emergence of high-stiffness filaments, improved layer adhesion, and more precise extrusion methods, the ability to print high-performance sensors will become increasingly viable. Establishing a rigorous testing and validation framework now ensures that a mature methodology is in place to support these technological advancements at the industrial readiness stage.

## Figures and Tables

**Table 1:** Material and Physical Properties for Simulation.

Property	Value
Density, ρ	1240 kg/m^3^
Young’s modulus, E	275 × 10^7^ N/m^2^
Mean radius, r	75 mm
Radial thickness, h	1 mm
Thickness-to-Radius Ratio, h/r	0.02

**Table 2:** Results summary.

Software	Method	$\omega_{n}$ (Hz)	ζ (%)	Q Factor
MEscope	Polynomial	47.07	0.4129	121.1
MATLAB	HPBW	47.11	0.5299	94.35
MATLAB	Exp Decay Fit	47.10	0.5068	98.65
MATLAB	FFT + SDOF Fit	47.10	0.5275	94.79

## Data Availability

The raw data supporting the conclusions of this article will be made available by the authors on request.
